# Aquaphotomics study of fresh cannabis inflorescence: near infrared spectral analysis of water matrix structures

**DOI:** 10.1007/s00216-024-05685-z

**Published:** 2024-12-09

**Authors:** Matan Birenboim, Nimrod Brikenstein, David Kenigsbuch, Jakob A. Shimshoni

**Affiliations:** 1 Department of Food Science, Institute for Postharvest and Food Sciences, Agricultural Research Organization, Volcani Center, P.O. Box 15159, 7505101 Rishon LeZion, Israel; 2 Department of Plant Science, The Robert H Smith Faculty of Agriculture, Food and Environment, The Hebrew University, 7610001 Rehovot, Israel; 3 Department of Postharvest Science, Institute for Postharvest and Food Sciences Agricultural Research Organization, Volcani Center, 7505101 Rishon LeZion, Israel

**Keywords:** Wet *Cannabis sativa* L. inflorescence, Fourier transform near-infrared spectroscopy (FT-NIR), Aquaphotomics, Water spectral pattern (WASP)

## Abstract

Aquaphotomics is an approach that describes the water–light interactions in aqueous solutions or biological systems and retrieves information about the nature of the underlying water-related interactions. We evaluated the water spectral pattern (WASP) and water matrix structure of freshly harvested cannabis inflorescence from seven different chemovars using near-infrared (NIR) spectral data coupled with chemometric models. Six activated water bands—1342, 1364, 1384, 1412, 1440, and 1462 nm, occurred consistently in all of the spectrum exploration steps as well as in the partial least squares-discriminant analysis (PLS-DA) steps. However, according to major class and chemovar aquagram values, the largest spectral variation was associated with the following bands: 1412, 1364, 1374, 1384, 1488, and 1512 nm. A strong positive correlation between 1364, 1374, and 1384 nm aquagram values and a strong negative correlation between 1412 and 1512 nm aquagram values were observed through all aquagram analysis steps. These water activated bands were found to serve as good discriminators and classifiers according to either major class or chemovar. Furthermore, significant differences in the water matrix structure of different cannabis chemovars were observed, with the highest variations associated with the presence of free water molecules, small molecule solvation shells, extent of strongly bound water, and the number of hydrogen bonds per water molecule. Minor cannabinoids and terpenes such as cannabigerolic acid and (-)-guaiol displayed relatively high correlations with these bands. The results of this study suggest that the most accurate way to explore the cannabis inflorescence water matrix spectral pattern is by chemovars and not by major classes.

**Graphical Abstract:**

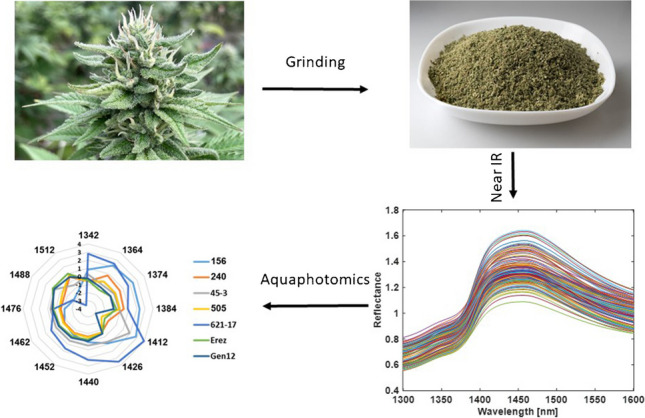

**Supplementary Information:**

The online version contains supplementary material available at 10.1007/s00216-024-05685-z.

## Introduction

*Cannabis sativa* L., an annual flowering plant belonging to the family *Cannabaceae*, is currently recognized as a single species (*Cannabis sativa* L.) [1–3]. Cannabis-based products have demonstrated therapeutic benefits for various medical conditions, including sleep disorders, nausea, anorexia, emesis, pain, inflammation, neurodegenerative disorders, epilepsy, and cancer [4–12]. The plant’s therapeutic potential is primarily attributed to its secondary metabolites, particularly cannabinoids and terpenes, which exceed 120 distinct compounds [13–15]. Notably, terpenes have been shown to enhance the effects of cannabinoids via the “entourage effect,” amplifying their pharmacological properties [13–15].

Recent years have seen a shift in the classification of cannabis cultivars, with the term “chemovar” gaining preference due to its focus on the full cannabinoid and terpene profile [16–18]. This approach accommodates the high chemical variability among modern cannabis cultivars [16–18]. Cannabis chemovars are broadly categorized into three major classes based on the ratio of the primary cannabinoids, (-)-Δ9-trans-tetrahydrocannabinolic acid (THCA) and cannabidiolic acid (CBDA): high THCA chemovars (≥ 10% dry weight [DW] of THCA), high CBDA chemovars (≥ 10% DW of CBDA), and hybrid chemovars (4–10% DW of both THCA and CBDA) [16, 19–22].

Near-infrared (NIR) spectroscopy has emerged as a rapid, accurate, and cost-effective analytical method widely employed for analyzing samples containing organic compounds with diverse functional groups [23–25]. NIR spectroscopy utilizes the near-infrared (700–1100 nm) and short-wave infrared (SWIR) (1100–2500 nm) regions of the electromagnetic spectrum [24, 26–28]. Recently, this method has been demonstrated to constitute a viable alternative to chromatography-based techniques for analyzing cannabinoids and terpenes in cannabis inflorescence [21, 29–35]. Most studies to date have focused on dry cannabis inflorescence, as water absorption bands within the NIR spectrum of wet cannabis inflorescence were considered a significant challenge for spectroscopic analysis due to the masking effects of water absorption bands in the NIR spectrum [28, 31, 36]. Specifically, water’s first overtone of the OH stretching band (1400–1550 nm) and the combination of OH stretching and bending bands (1850–2100 nm) frequently dominate spectral regions crucial for the prediction of various secondary metabolites [28, 31, 36]. Notwithstanding, recently we have demonstrated the feasibility of NIR spectroscopy for classifying cannabis chemovars and predicting the concentrations of cannabinoids and terpenes in wet cannabis inflorescence [37].

Freshly harvested cannabis inflorescence contains substantial water content, typically ranging from 75 to 80%, depending on the chemovar [17, 37]. Recent emerging research has revealed that water molecules in biological matrices are not merely passive solvents but active participants in the structural and functional dynamics of the system [36, 38–40]. Water interacts with organic and ionic compounds, forming various bonds such as hydrogen bonds, ionic bonds, and solvation shells [36, 38–40]. These interactions influence the physicochemical properties and stability of bioactive compounds, including cannabinoids and terpenes, within cannabis inflorescence. Despite the pivotal role of water in biological systems, the specific interactions between water and cannabinoids or terpenes have not been thoroughly investigated. Furthermore, the potential of water spectral patterns (WASP) to elucidate unique water matrix structures associated with different cannabis chemovars remains largely unexplored.

Aquaphotomics provides a novel approach to addressing these challenges. This methodology focuses on the interaction of water with light in the NIR spectrum, characterizing water matrix structures and their variations [36, 38, 41, 42]. By examining the spectral signatures of water, aquaphotomics captures the “molecular mirror” effect, reflecting the complex interactions between water molecules and other chemical constituents [36, 38].

The first overtone region of water’s OH stretching band (1300–1600 nm) encompasses 12 specific water vibration bands, termed Water Matrix Coordinates (WAMACS), which are associated with various states of water, including free water molecules, water dimers, hydration shells, and water molecules with varying numbers of hydrogen bonds [36, 38, 41]. Aquaphotomic studies in biological systems such as honey, cheese, watermelon, apples, soybeans, and papaya leaves have provided valuable insights into the interactions between water and dissolved compounds [36, 38–41, 43–45]. However, to the best of our knowledge, no similar study has been conducted on wet cannabis inflorescence.

This study aims to explore the potential of aquaphotomics in combination with NIR spectroscopy for analyzing wet cannabis inflorescence from seven distinct chemovars. Specifically, the objectives include (1) classifying cannabis samples based on major classes and chemovars, (2) investigating and comparing water matrix structures across chemovars, and (3) examining the relationships between the chemical composition of cannabis samples and their respective water matrix structures. Through this work, we seek to enhance the understanding of water’s role in cannabis inflorescence and develop robust prediction models for cannabinoids and terpenes.

## Materials and methods

### Chemicals

Acetonitrile, anhydrous ammonium formate, ethanol, and formic acid were obtained from Sigma-Aldrich (HPLC grade, Saint Louis, MO, US). Ultra-pure water was provided by the Milli-Q Plus system (Millipore Corp., Billerica, MA, USA). Cannabinoid analytical standards were purchased from RESTEK (RESTEK, Bellefonte, PA, USA): cannabidivarinic acid (CBDVA), cannabigerovarinic acid (CBGVA), cannabidiolic acid (CBDA), cannabigerolic acid (CBGA), cannabigerol (CBG), cannabidiol (CBD), (-)-Δ9-trans-tetrahydrocannabivarin (THCV), (-)-Δ9-trans-tetrahydrocannabivarinic acid (THCVA), cannabinol (CBN), cannabinolic acid (CBNA), cannabichromevarinic acid (CBCVA), (-)-Δ9-trans-tetrahydrocannabinol (Δ−9-THC), (-)-Δ8-trans-tetrahydrocannabinol (Δ−8-THC), cannabicyclol (CBL), cannabicyclolic acid (CBLA), cannabichromene (CBC), (-)-Δ9-trans-tetrahydrocannabinolic acid (THCA), and cannabichromenic acid (CBCA). Each of those standards was obtained at a stock concentration of 1000 µg/mL except CBLA which was obtained at a stock concentration of 500 µg/mL. Terpene standard mix at a stock concentration of 2500 µg/mL from each terpene contains the following terpenes: α-pinene, camphene, (-)-β-pinene, β-myrcene, δ−3-carene, α-terpinene, p-cymene, d-limonene, ocimene, γ-terpinene, terpinolene, linalool, (-)-isopulegol, geraniol, β-caryophyllene, α-humulene, nerolidol, (-)-guaiol, and (-)-α-bisabolol, which were obtained from RESTEK (RESTEK, Bellefonte, PA, USA).

### Plant material

Fresh (wet) medicinal *Cannabis sativa* L. inflorescences from seven different commercially available chemovars, namely, 621–17, Erez, 505, and 240 (high THCA chemovars), Gen12 (hybrid chemovar), and 156 and 45–3 (high CBDA chemovars), were provided by the Bar-lev farm between February 2023 and November 2023 (Bar-Lev Agricultural Crops, Kfar Hess, Israel, 32°15′21.2″N 34°57′01.0″E). All chemovars were analyzed on the same day of harvest for their cannabinoid and terpene content at the Agricultural Research Organization, the Department of Food Science, Israel. The growing conditions for the different cannabis chemovars are detailed in our previous publication [46].

The moisture content of each chemovar was assessed according to the procedure published in Birenboim et al. by subjecting the cannabis inflorescence to drying at a temperature of 105 °C in a dry oven (Model DFO-150, MRC, Harlow, UK, Table S1) [46].

### Sample preparation

The sample preparation process was carried out as described in Birenboim et al. [37]. In brief, for each chemovar, 5–7 inflorescences obtained from different plants of the same chemovar, weighing a total of 15–20 g, were homogenously ground (each inflorescence was ground separately) using a mortar and pestle with liquid nitrogen, yielding 20–30 samples per chemovar and a total of 187 samples [37]. Each sample (approximately 500 mg) was inserted into a glass vial and directly analyzed using the FT-NIR spectrometer (Thermo Fisher Antaris II FT-NIR Analyzer, Thermo Fisher Scientific, Waltham, MA, USA) [37]. For the determination of cannabinoid and terpene levels, the same homogenously ground cannabis samples (95–105 mg) used for the FT-NIR spectra measurement were extracted with 4 mL of ethanol in 15-mL Falcon tubes and shaken (Digital Orbital Shaker, MRC, Israel) in the dark for 15 min at 500 rpm [37]. One milliliter of the extract was transferred to an Eppendorf tube and centrifuged for 4 min at 12,000 rpm [37]. Subsequently, 0.75 mL of the supernatant was inserted into a HPLC vial and subjected to HPLC-photodiode-arrays (HPLC–PDA) analysis and GC/MS analysis for the elucidation of the cannabinoid and terpene composition, respectively [37].

### Quantification of cannabinoids by HPLC–PDA and terpenes by GC/MS

The ethanolic cannabis extracts were analyzed as described in Birenboim et al., utilizing HPLC–PDA (Acquity Arc FTN-R; Model PDA-2998, Waters Corp., Milford, MA, USA) equipped with Kinetex® 1.7 μm XB-C18 100A LC column (150 × 2.1 mm i.d. and 1.7 μm particle size; Phenomenex, Torrance, CA, USA) for the cannabinoids analysis [22]. Cannabinoids were quantified by comparing the integrated peak area with the corresponding cannabinoid calibration curve ranging from 1 to 1000 µg/mL [22]. The terpene analysis was carried out by GC/MS (Agilent, Santa Clara, CA, USA) as recently reported by Birenboim et al. utilizing DB-5 capillary column (5% phenyl, 95% dimethylpolysiloxane, 30 m × 0.250 mm, 0.25 m; Agilent, Santa Clara, CA, USA) for analyte separation [22]. Terpenes were quantified by comparing the integrated peak area with the corresponding terpene calibration curve ranging from 0.5 to 250 µg/mL [22]. The methods’ analytical validation parameters (i.e., *R*^2^, limit of detection, limit of quantification, repeatability, and accuracy) were recently published in Birenboim et al. [22]. For calculating the cannabinoid and terpene content, the cannabis wet inflorescence weight was normalized to dry weight using the average wet water content of each chemovar (Table S1), and the concentrations of the different compounds were reported in dry wt% (DW%) [46].

### Sample FT-NIR measurements

The FT-NIR spectral data was obtained using a Thermo Fisher Antaris II FT-NIR Analyzer (Thermo Fisher Scientific, Waltham, MA, USA) equipped with an integrated sphere and indium gallium-arsenic (In-Ga-As) detector as described in Birenboim et al. [21]. In brief, the reflectance spectra were measured with a resolution of 4 cm^−1^ in the range of 1000–2500 nm. A total of 16 scans were performed for each measurement, and each sample was measured four times from different directions. Spectral absorbance values were recorded in reflectance mode as log 1/*R*, where *R* is the sample reflectance. For aquaphotomics purposes, only the region of the first overtone of the OH stretching band (1300–1600 nm) was used. The NIR spectra were acquired under tightly controlled temperature conditions of 23 ± 1 °C, maintained using an air conditioning system (Elektra, Israel).

### Chemometrics

Spectrum exploration and the identification of significant bands within the first overtone of the OH stretching band was applied using a preprocessing transformation (PPT) of second derivative followed by multiplicative scatter correction (MSC) and smoothing in order to overcome overlapping peaks and discover major differences between chemovars or major classes [38, 47].

Next, partial least squares-discriminant analysis (PLS-DA) classification models according to major classes (i.e., high THCA, high CBDA, and hybrid) and chemovars (i.e., 621–17, Erez, 505, 240, Gen12, 156, and 45–3) were used to discover additional significant bands using the latent variables (LVs) loadings and the regression vectors of the models [38, 39, 42]. A PPT was applied as a crucial first model-development step, according to the commonly used PPT procedure in this research field [38–40, 42]. We applied several PPTs to the raw data, including first and second derivatives (1st and 2nd derivative, respectively), detrend, standard normal variate (SNV), MSC, smoothing, mean centering normalization method, and their possible combinations [21, 36, 38–40, 42, 48–52]. After applying the aforementioned methods, we determined that the optimal PPT for each model according to sensitivity, specificity, and accuracy values was MSC followed by smoothing.

PLS-DA was performed using 187 samples from the seven different aforementioned chemovars and their corresponding FT-NIR spectra as previously described in Birenboim et al. [21, 37]. In brief, the PLS-DA model was cross-validated using the Venetian blinds method with ten splits, followed by an independent prediction test group (i.e., *n* = *125* for the calibration/cross-validation group and *n* = *62* for the independent prediction group). The samples from each chemovar were randomly assigned to calibration and prediction groups using MATLAB 2021a (MathWorks Inc., MA, USA) software, maintaining a ratio of 67 to 33%, respectively. We evaluated the performance of the model as described in our previous publications, using sensitivity, specificity, and accuracy values [21, 22]. PLS-DA was performed using MATLAB 2021a (MathWorks Inc., MA, USA) and PLS_Toolbox 9.2 (Eigenvector, WA, USA).

### Aquaphotomics

The water spectral pattern (WASP) of the different major classes and chemovars was analyzed using aquagram for the 12 WAMACS of the first overtone of the OH stretching band [36, 38]. The normalized value for each major class or chemovar was calculated as follows [36, 38]:

$${A}_{\lambda }^{{\prime}}=\frac{{A}_{\lambda }-{\mu }_{\lambda }}{{\sigma }_{\lambda }}$$

where A′_λ_ is the normalized value of each WAMAC, *A*_λ_ is the value after MSC scatter correction, *μ*_λ_ is the mean value of all spectra assigned for specific group of samples, and *σ*_λ_ is the standard deviation value of all spectra assigned for specific group of samples [36, 38].

Pearson *r* correlation coefficients matrix between the major classes aquagram or chemovar aquagram values of the different WAMACS were calculated using GraphPad PRISM 10 (San Diego, CA, USA). Moreover, Pearson *r* correlation coefficients values between the chemovar aquagram WAMACS values and the chemical composition of the different chemovars were calculated using GraphPad PRISM 10 (San Diego, CA, USA).

Principal component analysis (PCA) according to the major class aquagram or chemovar aquagram values of the different WAMACS was performed using MATLAB 2021a (MathWorks Inc., MA, USA) and PLS_Toolbox 9.2 (Eigenvector, WA, USA). The first two principal components (PCs) scores and loadings were plotted and analyzed.

### Statistical analysis

For each compound analyzed, two-way ANOVA was used to determine the differences in cannabinoid and terpene concentrations between the different chemovars, at *α* = 0.05 using GraphPad PRISM 10 (San Diego, CA, USA).

Moreover, for each aquagram WAMAC, two-way ANOVA was used to determine the differences in aquagram values of the different major classes or chemovars, at *α* = 0.05 using GraphPad PRISM 10 (San Diego, CA, USA).

## Results and discussion

### Spectrum exploration

**Fig. 1:**
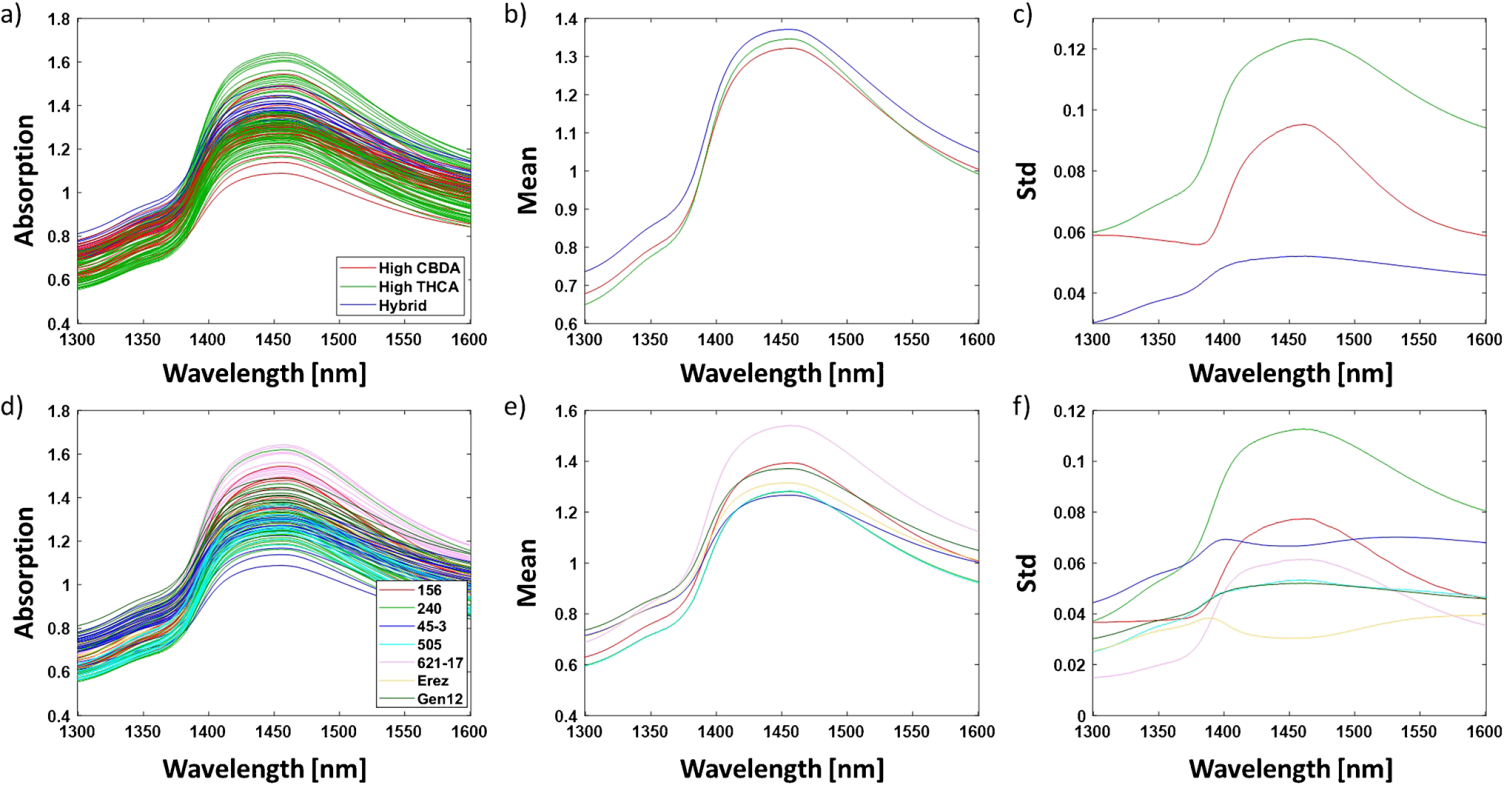
FT-NIR raw reflectance spectra in the wavelength region of 1300–1600 nm of the complete set of samples labeled according to **a** major classes and **d** chemovars. Additionally, **b** the mean spectra and **c** standard deviation of the different major classes and **e** the mean spectra and **f** standard deviation of the different chemovars are provided

The analysis and spectrum exploration were performed on the OH first overtone in the region of 1300–1600 nm, according to the guidelines published in Tsenkova et al. (Fig. 1) [38]. Averaging the spectral data of samples according to either major classes or chemovars was performed to minimize the influence of variations not of primary interest, such as humidity levels [38]. This approach aimed to uncover significant differences between the sample groups concerning the interactions of the water matrix with the secondary metabolites of cannabis inflorescence [38]. The hybrid major class displayed the highest average reflectance values in the entire region of the OH first overtone, while the high CBDA major class exhibited larger average reflectance values than the high THCA major class in the region of 1300–1380 nm and 1550–1600 nm (Fig. 1b). On the other hand, the latter displayed larger average reflectance values in the region of 1380–1550 nm as compared to the high CBDA major class (Fig. 1b). The chemovar analysis revealed that the 621–17 chemovar displayed the largest average reflectance values in the region of 1380–1600 nm, while the 240 and 505 chemovars had the lowest average reflectance values in the entire region of the OH first overtone (Fig. 1e). As revealed by the standard deviation plots, the highest variation in most major classes and chemovars is observed at 1450 nm (C8, Fig. 1c and f) [36].

**Fig. 2:**
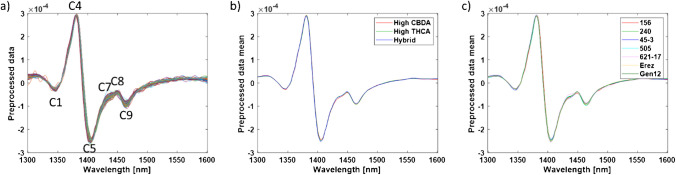
Preprocessed FT-NIR reflectance spectra using 2nd derivative followed by MSC and smoothing in the wavelength region 1300–1600 nm of **a** the complete set of samples and the mean preprocessed FT-NIR reflectance spectra of **b** major classes and **c** chemovars

After averaging, the raw data was preprocessed using 2nd derivative, followed by MSC and smoothing, in order to discover activated water bands which are not visible in the original averaged spectrum (Fig. 2) [38–41]. These spectrum exploration steps revealed several water absorbance bands activated in the different cannabis chemovars, i.e., 1348 nm (C1), 1382 nm (C4), 1404 nm (C5), 1433 nm (C7), 1449 nm (C8), and 1466 nm (C9) [36]. The largest variation in the preprocessed spectrum for both major classes and chemovars was obtained at 1404 nm (C5), 1433 nm (C7), 1449 nm (C8), and 1466 nm (C9) (Fig. 2).

### PLS-DA classification

PLS-DA classification models were developed based on the OH first overtone spectral data to distinguish between major classes and chemovars. These models aimed to identify additional water-activated absorbance bands and evaluate whether classification can be achieved using the OH first overtone spectrum only [38, 39, 42]. To reveal such bands, the loadings and regression vectors of these classification models were analyzed following the guidelines established by Tsenkova et al. [38].

The PLS-DA classification yielded an excellent major class separation and prediction model, utilizing eight LVs (Table 1). The calibration, cross-validation, and prediction groups displayed high sensitivity, specificity and accuracy values (> 0.94, Table 1). Only two misclassifications of the high CBDA major class were observed in the cross-validation data set, whereas no misclassifications were obtained for the independent prediction group. Consequently, sensitivity and specificity values approaching unity were obtained, indicative of highly accurate classification model [53]. The root mean square error of calibration (RMSEC), root mean square error of cross-validation (RMSECV), and root mean square error of prediction (RMSEP) values for the different major classes ranged between 0.190 and 0.241, and the RMSECV/RMSEC and RMSEP/RMSECV ratios were below 1.1, indicative of negligible model over-fitting (Table 1) [19, 54]. Overall, the PLS-DA model provided an excellent classification model of all cannabis major classes.

**Table 1:** Cross-validation and prediction performance parameters of the PLS-DA major class classification model of wet cannabis inflorescence samples

	High THCA	High CBDA	Hybrid
Total number of samples	56	101	30
Sensitivity (cross-validation)	0.946	1	1
Specificity (cross-validation)	1	0.983	0.990
Accuracy (cross-validation)	0.984	0.992	0.992
Sensitivity (prediction)	1	1	1
Specificity (prediction)	1	1	1
Accuracy (prediction)	1	1	1
RMSEC^a^	0.192	0.229	0.190
RMSECV^b^	0.204	0.241	0.203
RMSEP^c^	0.193	0.218	0.203

^a^*RMSEC*, root mean square error of calibration. ^b^*RMSECV*, root mean square error of cross-validation. ^c^*RMSEP*, root mean square error of prediction

The chemovar PLS-DA classification model utilizing seven LVs yielded good separation and good chemovar predictive capability (Table 2). The calibration, cross-validation, and prediction groups displayed specificity and accuracy values higher than 0.96, while the sensitivity values were higher than 0.87, except for chemovar 156, which displayed a sensitivity value of 0.75 (Table 2). Furthermore, only eight samples, were misclassified in the cross-validation dataset, three of these sample belong to the 156 chemovar, whereas only one samples was misclassified in the independent prediction group. The RMSEC, RMSECV, and RMSEP values for chemovar classification ranged between 0.184 and 0.273, similar to the corresponding major class classification model values, indicating similar classification performances (Table 2). The RMSECV/RMSEC and RMSEP/RMSECV ratios were below 1.1, indicative of negligible model over-fitting to the data (Table 2). Taken altogether, the classification models provided good to excellent major class and chemovar classifications, and therefore, the loadings and regression vectors of these models were used to further reveal additional water activated absorbance bands.

**Table 2:** Cross-validation and prediction performance parameters of the PLS-DA chemovar classification model of wet cannabis inflorescence samples

	621–17	Erez	505	240	Gen12	156	45–3
Major class	High THCA	High THCA	High THCA	High THCA	Hybrid	High CBDA	High CBDA
Total number of samples	21	30	25	25	30	26	30
Sensitivity (cross-validation)	0.928	0.950	0.875	0.882	1	0.750	1
Specificity (cross-validation)	0.982	1	0.991	0.972	0.981	0.991	0.990
Accuracy (cross-validation)	0.976	0.992	0.976	0.960	0.984	0.960	0.992
Sensitivity (prediction)	1	1	1	1	0.889	1	1
Specificity (prediction)	1	1	1	0.981	1	1	1
Accuracy (prediction)	1	1	1	0.984	0.984	1	1
RMSEC^a^	0.219	0.194	0.210	0.263	0.238	0.248	0.204
RMSECV^b^	0.229	0.202	0.218	0.273	0.250	0.256	0.212
RMSEP^c^	0.234	0.184	0.214	0.248	0.242	0.252	0.198

^a^*RMSEC*, root mean square error of calibration. ^b^*RMSECV*, root mean square error of cross-validation. ^c^*RMSEP*, root mean square error of prediction

**Fig. 3:**
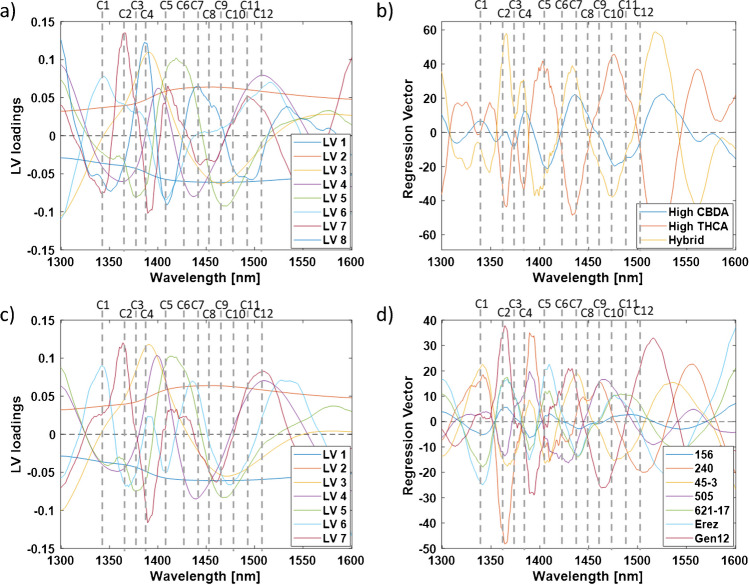
**a** LV loadings and **b** regression vectors plots of major class PLS-DA classification model and **c** LV loadings and **d** regression vectors plots of chemovar PLS-DA classification model. The dashed grey lines represents the 12 different activated water bands in the first overtone region of the OH stretching ban

The bands that exhibited the highest local positive or negative contributions to the classification models, as indicated by the LV loadings and regression vector plots, were identified and recorded as water-activated absorbance bands (Fig. 3) [38]. Although the first two latent variables (LVs) accounted for over 99% of the data variation, it is essential to examine all LV loadings since subtle changes in the water matrix may be captured by higher-order LV loading vectors, which could provide additional significant information [38]. Summarizing the water activated absorbance bands obtained by each LV loadings and regression vector plots revealed that six activated water bands (i.e., C1, C2, C4, C5, C7, and C9) consistently occurred following the aquaphotomics analysis and, thus, may be considered as more informative than the other bands (Table S2) [36, 38]. Five of these bands (i.e., C1, C4, C5, C7, and C9) were also identified in the spectrum exploration step (Figs. 1 and 2). The identification of these bands is crucial for understanding the aqueous system and the water matrix structure of the different cannabis chemovars, as illustrated in aquagrams in the following section [38].

### Aquaphotomics

**Fig. 4:**
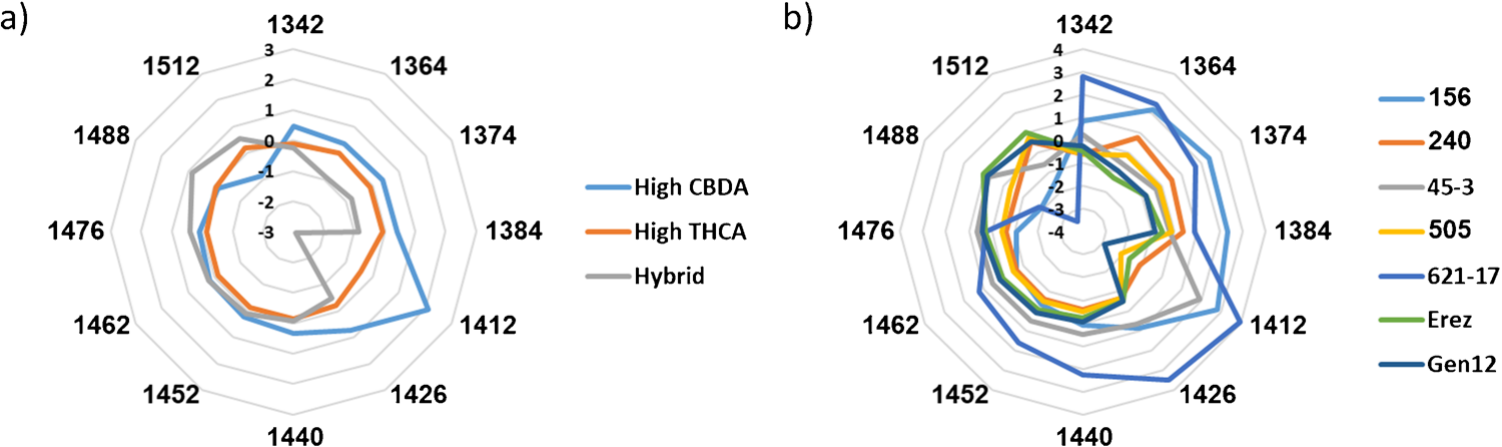
Aquagrams of the OH first overtone 12 WAMACS of **a** major classes and **b** chemovars

Aquagram values according to major classes and chemovars were calculated and plotted according to the guidelines published by Tsenkova et al. [36, 38]. Significant differences in the water spectral patterns were observed for both major classes and chemovars (Fig. 4).

In the major class aquagram, the highest variations were observed at 1412 nm (C5) followed by 1364, 1374, 1384 nm (C2–C4), and 1512 nm (C12, Fig. 4a). These water activated absorbance bands were also occurred in several major class LVs or regression vectors (Table S2).

The aquagram values at 1412 nm (C5) indicate that the high CBDA chemovars exhibit the highest presence of free water in the inflorescence matrix, followed by the high THCA and hybrid major classes (Fig. 4a, Table S3) [36, 38]. Furthermore, the aquagram values at 1364, 1384, 1426, and 1452 nm (C2, C4, C6, and C8, respectively) indicate that the high CBDA chemovars also possess the strongest water solvation shells and the highest water hydration levels (Fig. 4a, Table S3). In contrast, the hybrid chemovar exhibit the weakest water solvation shells and the lowest water hydration levels (Fig. 4a, Table S3) [36, 38].

Conversely, the aquagram values at 1512 nm (C12) reveal that the high CBDA chemovars exhibited the lowest amount of strongly bound water, whereas the hybrid major class displayed a high amount of strongly bound water (Fig. 4a, Table S3) [36, 38]. This observation is further supported by the aquagram values at 1440, 1462, 1476, and 1488 nm (C7, C9, C10, and C11, respectively), which indicate that the high CBDA chemovars displayed the highest number of water molecules with a single hydrogen bond (Fig. 4a) [36, 38]. In contrast, the hybrid major class revealed the highest number of water molecules with two, three, and four hydrogen bonds (Fig. 4a) [36, 38]. However, only 1488 nm aquagram values were found to be statistically significant (Table S3). These results are in agreement with the free water, water solvation, and water hydration results. For example, the high CBDA major class exhibited a significant presence of free water, strong water solvation shells, and high degree of water hydration and also revealed the lowest quantity of strongly bound water but a high number of hydrogen bonds.

Overall, the high THCA chemovars revealed the lowest number of hydrogen bonded water molecules (Fig. 4a). Notwithstanding, due to lower variations in the bands related to hydrogen bonded water molecules, it is reasonable to assume that these differences between the major classes are not that significant as compared to the free water differences (Fig. 4a, Table S3).

Examination of the chemovar aquagram reveals several significant differences and similarities compared to the major class aquagram. In both cases, the highest variation is observed at 1412 nm (C5), followed by 1364, 1374, 1384 nm (C2–C4), 1488 nm, and 1512 nm (C11–C12, Fig. 4). This indicates that these water-activated bands can serve as effective discriminators and classifiers for both major classes and chemovars, particularly the C2–C5 bands, which were consistently identified throughout the aquaphotomics analysis steps (Fig. 4, Table S2–S4). Thus, the aquagram of each chemovar can act as a unique fingerprint, as it reflects the dominant characteristics and relationships with the chemovar’s water matrix structure [39]. Additionally, the hybrid chemovar Gen12 in the dataset exhibited the lowest aquagram values at 1374, 1384, and 1412 nm (C3–C5), whereas the high CBDA chemovars demonstrated the highest aquagram values at 1374, 1384, and 1476 nm (C3, C4, and C10, respectively), consistent with the major class aquagram values (Fig. 4b, Table S4).

On the other hand, the high CBDA and high THCA chemovars exhibit significant differences in their water spectral patterns (Fig. 4b). For example, the 156 chemovar had a much higher aquagram values at 1364, 1374, and 1384 nm (C2–C4) and much lower values at 1488 nm (C11) compared to the 45–3 chemovar, despite both belonging to the high CBDA major class (Fig. 4b, Table S4). Conversely, both high CBDA chemovars showed similar aquagram values at 1342, 1412, 1426, 1440, and 1512 nm (C1, C5, C6, C7, and C12, respectively, Fig. 4b, Table S4). In the case of the high THCA chemovars, the 621–17 chemovar presented much higher aquagram values at 1342, 1364, 1412, 1426, 1452, and 1462 nm (C1, C2, C5, C6, C8, and C9, respectively) and much lower values at 1512 nm (C12) compared to the other three high THCA chemovars (Fig. 4b, Table S4). The aquagram values for the 240, 505, and Erez chemovars were similar, except for the 1364 and 1488 nm (C2 and C11) values in the 240 and Erez chemovars (Fig. 4b, Table S4). It is important to note that adding more chemovars from different major classes to the dataset may reveal additional chemovars with trends differing from their major class aquagram. This is because the classic aquagram is a relative construct that depends on the samples in the dataset and their affiliations [38]. These results suggest that chemovars within the same major class may have completely different water matrix structures.

According to the aquagram values at 1412 nm (C5), the 621–17 chemovar exhibited the highest presence of free water in the inflorescence matrix, followed by both high CBDA chemovars (Fig. 4b, Table S4) [36, 38]. In contrast, the hybrid chemovar, Gen12, had the lowest presence of free water (Fig. 4b, Table S4) [36, 38]. The aquagram values at 1364 and 1384 nm (C2 and C4, respectively) suggested that the 621–17 and 156 chemovars had strong water solvation shells of small molecules, while the Erez and Gen12 chemovars had weak water solvation shells of small molecules (Fig. 4b, Table S4) [36, 38]. Furthermore, the aquagram values at 1426 nm (C6) indicated that the 621–17 chemovar had the highest water hydration, whereas the hybrid chemovar Gen12 and the other three high THCA chemovars—505, 240, and Erez—exhibited the lowest water hydration (Fig. 4b, Table S4) [36, 38].

On the other hand, the Erez chemovar, followed by the 505, 240, and Gen12 chemovars, had the highest amount of strongly bound water, while the 621–17 chemovar had the lowest amount of strongly bound water, as suggested by the 1512 nm aquagram values (Fig. 4b, Table S4) [36, 38]. The Erez chemovar also had the highest number of water molecules with four hydrogen bonds, whereas the 621–17 chemovar had the highest number of water molecules with one and two hydrogen bonds (Fig. 4b, Table S4) [36, 38]. The 1476 nm (C10) aquagram values, which are not statistically significant, suggested that the water matrices of all chemovars contained a similar number of water molecules with three hydrogen bonds (Fig. 4b, Table S4) [36, 38]. The 240 and 505 chemovars had the lowest number of water molecules with one and two hydrogen bonds, while the 156 chemovar had the lowest number of water molecules with four hydrogen bonds (Fig. 4b, Table S4 [36, 38].

These findings revealed that within each major class, chemovars exhibited different water spectral patterns, with the most significant variations related to the presence of free water, solvation shells of small molecules, the amount of strongly bound water, and the number of hydrogen bonds (Fig. 4b) [36, 38]. These results suggest that the most accurate way to explore the water matrix spectral patterns of cannabis inflorescence is by chemovar rather than by major class.

Although the C7 and C9 bands were consistently observed in the previous steps of the aquaphotomics analysis, they exhibited lower variation in their aquagram values (Fig. 4, Table S2–S4). This suggests that the number of water molecules with one and two hydrogen bonds is relatively similar across different chemovar water matrix structures, with the exception of the 621–17 chemovar (Table S4) [36, 38]. In contrast, the aquagram values of the C11 and C12 bands showed high variations, indicating that one of the main differences in the water matrix structures is related to strongly bound water with four hydrogen bonds (Fig. 4, Table S2–S4) [36, 38].

**Fig. 5:**
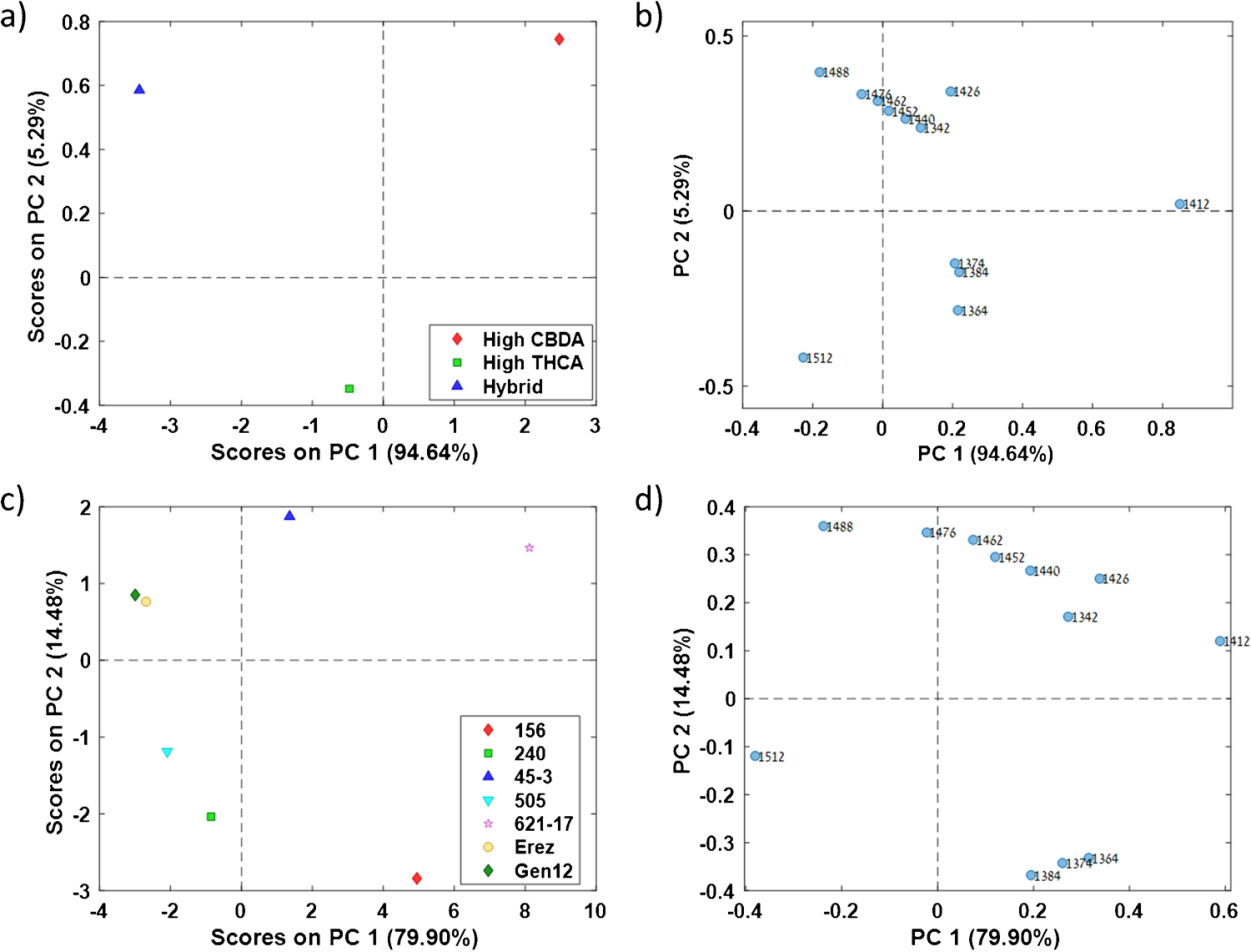
PCA analysis of the OH first overtone 12 WAMACS values. Major classes first two PCs **a** score and **b** loading and chemovars first two PCs **c** score and **d** loading plots

To further evaluate the cannabis inflorescence water matrix spectral pattern, we used PCA analysis and Pearson *r* correlation coefficients matrix of the WAMACS aquagram values, to reveal major correlations between different WAMACS and chemovars. Positively correlated bands can be identified based on their vectors direction, where smaller angle between two vectors suggests stronger positive correlation and angle approaching 180° suggests stronger negative correlation [22, 48, 55]. The high CBDA major class found to positively correlate to 1342, 1426, and 1440 nm (C1, C6, and C7, respectively) aquagram values, the high THCA major class found to positively correlate to 1512 nm (C12) aquagram values, while the hybrid major class found to positively correlate to 1488 nm (C11) aquagram values (Fig. 5a and b). The 621–17 chemovar was found to positively correlate with the 1412 nm (C5) aquagram values, while the 45–3 chemovar positively correlated with the 1452 and 1462 nm (C8–C9) aquagram values (Fig. 5c and d). The Gen12 and Erez chemovars showed positive correlations with the 1488 nm (C11) aquagram values, and the 505 chemovar positively correlated with the 1512 nm (C12) aquagram values (Fig. 5c and d). The 156 chemovar exhibited positive correlations with the 1364, 1374, and 1384 nm (C2–C4) aquagram values (Fig. 5c and d). These findings are consistent with the original chemovar aquagram values (Fig. 4b).

According to both major class and chemovar PCA loadings, there is a positive correlation among the 1364, 1374, and 1384 nm (C2–C4) aquagram values and a negative correlation between the 1412 and 1512 nm (C5 and C12, respectively) aquagram values (Fig. 5b and d). These high correlations were also observed in the Pearson correlation coefficient matrix for both major class and chemovar, reinforcing the PCA loadings results as a reliable tool for identifying correlated water-activated bands (Figure S1). These findings align with the chemical interpretation of these water-activated bands, 1364 and 1384 nm (C2 and C4, respectively), are associated with water solvation shells, while 1412 and 1512 nm (C5 and C12, respectively) correspond to free water molecules and strongly bound water molecules, respectively [36, 38].

The Gen12 and Erez chemovars exhibited similar PCA scores despite belonging to different major classes (Fig. 5c). Furthermore, the 621–17 chemovar showed a positive PC1 score, whereas all other high THCA chemovars had negative PC1 scores (Fig. 5c). These results are consistent with the chemovar aquagram values and further support the observation that the water matrix structure of each chemovar is distinct.

### Aquagram and chemical composition correlation

The final step was investigating the relationship between chemovar aquagram values and the different chemovars chemical composition [41]. For that purpose, Pearson *r* correlation coefficients between each WAMAC and major active compound concentrations were calculated (Table 3) [41]. The concentrations of major cannabinoids and terpenes are detailed in Table S5. Pearson *r* correlation coefficients values varied between − 0.81 and 0.75 (Table 3). The major cannabinoids, i.e., THCA and CBDA, alongside CBCA, as well as the terpenes β-myrcene, β-caryophyllene, and α-humulene concentrations, revealed poor correlations to all aquagram values (Table 3). These results suggest that there is no clear relationship between the major compound concentrations to the cannabis inflorescence water matrix structure. On the other hand, CBGA and (-)-guaiol displayed relatively high correlations (higher than 0.68 and lower than − 0.75) to various aquagram values (Table 3). CBGA exhibited a strong negative correlation with C5, which represents free water molecules, and a strong positive correlation with C12, indicative of strongly bound water (Table 3) [36, 38]. This suggests that higher CBGA concentrations result in a water matrix with a greater amount of strongly bound water compared to free water molecules (Table 3) [36, 38]. It is important to note that CBGA is also the precursor for the three main acidic cannabinoids: THCA, CBDA, and CBCA [3, 14, 56]. Consequently, understanding these correlations is crucial for elucidating the metabolic pathways in each of the chemovars. Despite the relatively low concentrations of (-)-guaiol in all chemovars compared to other terpenes (Table S3), it exhibited a strong positive correlation with C2–C4, which are associated with water solvation shells, and a strong negative correlation with C10 and C11, which are related to water molecules with a high number of hydrogen bonds (Table 3) [36, 38]. This indicates that higher (-)-guaiol concentrations result in a water matrix with fewer strongly bound water molecules (Table 3). The correlation of (-)-guaiol concentrations to five different water activated bands supports the hypothesis that small perturbation in the water matrix affects the various water molecules conformations, as the differences in (-)-guaiol concentrations are minor as compared to the other cannabinoids and terpenes [38]. Moreover, α-pinene, (-)-β-pinene, and d-limonene exhibited positive or negative moderate correlations (higher than 0.5 and lower than − 0.5) to various aquagram values (Table 3). These results suggest that compounds present at low concentrations might influence the water matrix structure even more than the major cannabinoids. In the case of aquagram values, all examined bands showed at least one moderate positive or negative correlation with at least one compound (Table 3). Specifically, C2–C5 and C10–C12 exhibited the most pronounced correlations, indicating that water solvation shells, free water, and strongly bound water have the greatest impact on the water matrix structure and aquagram pattern of cannabis inflorescence [36, 38].

**Table 3:**
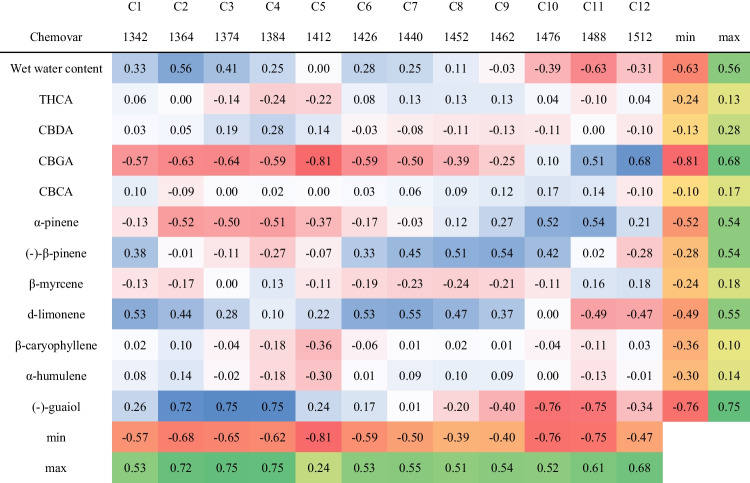
Pearson *r* correlation coefficients values between the chemovar aquagram WAMACS values and the chemical composition/wet water content of the different chemovars

The water matrix structure, as indicated by aquagram values, can reflect the presence and interactions of various secondary metabolites, such as cannabinoids and terpenes, which are crucial for the medicinal properties of cannabis. For instance, chemovars with higher free water content, indicated by aquagram values at 1412 nm, might correlate with higher concentrations of certain cannabinoids that enhance solubility in water, potentially influencing their bioavailability and therapeutic effects. Similarly, the presence of strong water solvation shells (e.g., 1364, 1374, 1384 nm) can indicate higher levels of smaller molecules like terpenes, which contribute to the entourage effect, enhancing the medicinal efficacy of cannabinoids. Thus, by analyzing the water matrix through aquaphotomics, researchers can gain insights into the chemical composition and potential medicinal benefits of different cannabis chemovars, allowing for a more targeted selection of chemovars for specific therapeutic purposes.

Although the aquaphotomics approach holds great promise for analyzing wet cannabis inflorescence, it comes with notable limitations. Aquaphotomics is highly sensitive to environmental factors, including temperature, and sample handling conditions. Variations in these parameters can influence the water matrix structure and affect reproducibility, requiring stringent experimental controls that may not be feasible in field or non-laboratory settings. Another significant limitation stems from the complexity of water interactions with organic constituents. Disentangling these interactions to attribute specific spectral features to particular compounds or structural changes is challenging. Furthermore, the aquaphotomics approach also faces challenges due to its dependence on the dataset composition. A classic aquagram is a relative construct that depends on the samples in the dataset and their affiliations. As a result, conclusions about differences in water matrix structures are limited by the diversity and representativeness of the sample set. To obtain more detailed and comprehensive insights, it is crucial to include samples from additional chemovars. This requirement highlights the importance of building extensive, chemically diverse datasets for robust and generalizable results. The lack of standardized protocols for aquaphotomics in cannabis research presents a barrier to reproducibility. Differences in sample preparation, spectral acquisition, and data analysis methodologies across studies can lead to variability in results and limit the comparability of findings. Despite these limitations, aquaphotomics offers a unique and powerful perspective on the role of water in biological systems. Addressing these challenges through methodological advancements, larger datasets, and integration with complementary techniques will help unlock the full potential of this innovative approach for studying wet cannabis inflorescence.

## Conclusions

In conclusion, the spectrum exploration steps and the development of PLS-DA classification models revealed six consistently recurring activated water bands: 1342, 1364, 1384, 1412, 1440, and 1462 nm (C1, C2, C4, C5, C7, and C9, respectively). The C7 and C9 bands exhibited lower variation in their aquagram values, suggesting that different chemovar water matrix structures have a similar number of water molecules with one and two hydrogen bonds. The combination of NIR spectroscopy and aquaphotomics analysis demonstrated significant differences in the water matrix structure among various cannabis chemovars. Both major class and chemovar aquagrams showed high variation at 1412 nm (C5), followed by 1364, 1374, and 1384 nm (C2–C4) and 1488 nm and 1512 nm (C11–C12). Consequently, these water-activated bands can serve as effective discriminators and classifiers based on either major class or chemovar.

Additionally, the results indicate that chemovars within the same major class can have completely different water matrix structures. The highest variations were related to the presence of free water, solvation shells of small molecules, the amount of strongly bound water, and the number of hydrogen bonds. Among the high THCA chemovars, the 621–17 chemovar exhibited a significantly different water matrix structure compared to the other three high THCA chemovars examined. This study suggests that the most accurate way to explore the water matrix spectral patterns of cannabis inflorescence is by chemovar rather than by major class.

Throughout all aquagram analysis steps, a positive correlation was observed among the 1364, 1374, and 1384 nm aquagram values, and a negative correlation was noted between the 1412 and 1512 nm aquagram values. No clear relationship was found between the concentrations of major compounds and the water matrix structure of cannabis inflorescence. Only CBGA and (-)-guaiol exhibited relatively high correlations with various aquagram values, while α-pinene, (-)-β-pinene, and d-limonene showed moderate correlations. Overall, the C2–C5 and C10–C12 bands demonstrated the most pronounced correlations with the chemical composition, implying that water solvation shells, free water, and strongly bound water have the highest impact on the water matrix structure and aquagram pattern of cannabis inflorescence. Taking altogether, analyzing the aquagrams of chemovars through aquaphotomics provides valuable insights into their chemical composition and potential medicinal properties, enabling a more targeted selection of chemovars for specific therapeutic applications.

## Supplementary Information

Below is the link to the electronic supplementary material.

Supplementary file1 (DOCX 380 KB)

## Data Availability

Data will be made available on request.

## Abbreviations

THCA: (-)-Δ9-*trans*-Tetrahydrocannabinolic acid
CBDA: Cannabidiolic acid
NIR: Near infrared
WAMACS: Water Matrix Coordinates
THC: (-)-Δ9-*trans*-Tetrahydrocannabinol
CBD: Cannabidiol
DW%: Dry weight%
CBDVA: Cannabidivarinic acid
CBGVA: Cannabigerovarinic acid
CBGA: Cannabigerolic acid
CBG: Cannabigerol
CBD: Cannabidiol
THCV: (-)-Δ9-*trans*-Tetrahydrocannabivarin
THCVA: (-)-Δ9-*trans*-Tetrahydrocannabivarinic acid
CBN: Cannabinol
CBNA: Cannabinolic acid
CBCVA: Cannabichromevarinic acid
Δ-9-THC: (-)-Δ9-*trans*-Tetrahydrocannabinol
Δ-8-THC: (-)-Δ8-*trans*-Tetrahydrocannabinol
CBL: Cannabicyclol
CBLA: Cannabicyclolic acid
CBC: Cannabichromene
CBCA: Cannabichromenic acid
HPLC-PDA: High-pressure liquid-chromatography-photodiode arrays
GC/MS: Gas chromatography-mass spectroscopy
PPT: Preprocessing transformation
MSC: Multiplicative scatter correction
PLS-DA: Partial least squares-discriminant analysis
LVs: Latent variables
WASP: Water spectral pattern
PCA: Principal component analysis
PCs: Principal components
RMSEC: Root mean square error of calibration
RMSECV: Root mean square error of cross-validation
RMSEP: Root mean square error of prediction
