# Supplementary material for: Aquaphotomics study of fresh cannabis inflorescence: near infrared spectral analysis of water matrix structures
Source: Anal Bioanal Chem. 2024 Dec 9;417(4):747–60. doi: 10.1007/s00216-024-05685-z (PMC11772404; doi:10.1007/s00216-024-05685-z)
Supplement: Supplementary file 1 — Supplementary file1 (DOCX 380 KB) [file 216_2024_5685_MOESM1_ESM.docx]

**Table S1.** Water content of wet cannabis inflorescence of each chemovar.

| **45-3** | **156** | **Gen12** | **240** | **505** | **Erez** | **621-17** | **Chemovar** |
| --- | --- | --- | --- | --- | --- | --- | --- |
| $73.4\pm2.1\%$ | $76.7\pm1.0\%$ | $77.4\pm1.6\%$ | $78.5\pm1.6\%$ | $78.5\pm2.0\%$ | $75\pm3\%$ | $79.7\pm0.4\%$ | Wet water content |

**Table S2.** WAMACS count summary according to spectrum exploration, PLS-DA LVs loadings and regression vectors.

|  | **C1** | **C2** | **C3** | **C4** | **C5** | **C6** | **C7** | **C8** | **C9** | **C10** | **C11** | **C12** |
| --- | --- | --- | --- | --- | --- | --- | --- | --- | --- | --- | --- | --- |
| **2nd der -> MSC -> smoothing** | **V** |  |  | **V** | **V** |  | **V** | **V** | **V** |  |  |  |
| **major class PLSDA PC1** |  |  |  |  |  |  |  |  | **V** |  |  |  |
| **major class PLSDA PC2** |  |  |  |  |  |  |  |  | **V** |  |  |  |
| **major class PLSDA PC3** |  |  |  | **V** |  |  |  |  | **V** |  |  |  |
| **major class PLSDA PC4** |  | **V** |  |  | **V** |  | **V** |  |  |  |  | **V** |
| **major class PLSDA PC5** |  | **V** | **V** |  |  | **V** |  |  | **V** |  |  |  |
| **chemovar PLSDA PC1** |  |  |  |  |  |  |  |  | **V** |  |  |  |
| **chemovar PLSDA PC2** |  |  |  |  |  |  |  |  | **V** |  |  |  |
| **chemovar PLSDA PC3** |  |  |  | **V** |  |  |  |  |  | **V** |  |  |
| **chemovar PLSDA PC4** |  | **V** |  |  | **V** |  | **V** |  |  |  |  | **V** |
| **chemovar PLSDA PC5** |  | **V** | **V** |  |  | **V** |  |  | **V** |  |  |  |
| **major class reg vec 1 (high CBDA)** | **V** | **V** | **V** | **V** | **V** |  | **V** |  |  | **V** |  |  |
| **major class reg vec 2 (high THCA)** | **V** | **V** | **V** | **V** | **V** |  | **V** |  |  | **V** |  |  |
| **major class reg vec 3 (hybrid)** | **V** | **V** | **V** | **V** | **V** |  | **V** |  |  | **V** |  |  |
| **chemovar reg vec 1 (156)** | **V** | **V** |  | **V** | **V** |  | **V** | **V** |  |  | **V** |  |
| **chemovar reg vec 2 (240)** | **V** | **V** |  | **V** | **V** |  |  |  | **V** |  |  | **V** |
| **chemovar reg vec 3 (45-3)** | **V** | **V** |  | **V** | **V** |  | **V** |  |  | **V** |  |  |
| **chemovar reg vec 4 (505)** | **V** | **V** |  | **V** |  | **V** |  |  | **V** |  |  | **V** |
| **chemovar reg vec 5 (621-17)** | **V** | **V** |  | **V** | **V** |  | **V** |  |  |  | **V** |  |
| **chemovar reg vec 6 (erez)** | **V** | **V** |  | **V** | **V** |  | **V** |  |  | **V** |  |  |
| **chemovar reg vec 7 (gen12)** | **V** | **V** |  | **V** |  | **V** |  |  | **V** |  |  | **V** |
| **count** | **10** | **14** | **5** | **13** | **11** | **4** | **10** | **2** | **11** | **6** | **2** | **5** |
| **count PC** | **0** | **4** | **2** | **2** | **2** | **2** | **2** | **0** | **7** | **1** | **0** | **2** |
| **count reg vec** | **9** | **10** | **3** | **10** | **8** | **2** | **7** | **1** | **3** | **5** | **2** | **3** |

**Table S3.** Two-way ANOVA results between major-class aquagram values at a significance value of p < 0.05. P value < 0.0332 (*), p < 0.0021 (**), p < 0.0002 (***), p < 0.0001 (****), Val, value.

|  | 1342 nm C1 | | 1364 nm C2 | | 1374 nm C3 | | 1384 nm C4 | | 1412 nm C5 | | 1426 nm C6 | | 1440 nm C7 | | 1452 nm C8 | | 1462 nm C9 | | 1476 nm C10 | | 1488 nm C11 | | 1512 nm C12 | |
| --- | --- | --- | --- | --- | --- | --- | --- | --- | --- | --- | --- | --- | --- | --- | --- | --- | --- | --- | --- | --- | --- | --- | --- | --- |
| Tukey's multiple comparisons test | Summary | P Val | Summary | P Val | Summary | P Val | Summary | P Val | Summary | P Val | Summary | P Val | Summary | P Val | Summary | P Val | Summary | P Val | Summary | P Val | Summary | P Val | Summary | P Val |
| high CBDA vs. high THCA | ns | 0.1007 | ns | 0.4269 | ns | 0.2115 | ns | 0.2115 | **** | <0.0001 | ** | 0.0019 | ns | 0.1844 | ns | 0.3457 | ns | 0.4704 | ns | 0.6997 | ns | 0.9288 | *** | 0.0002 |
| high CBDA vs. hybrid | ns | 0.1487 | ** | 0.0022 | ** | 0.0032 | ** | 0.0015 | **** | <0.0001 | ** | 0.0029 | ns | 0.4817 | ns | 0.9218 | ns | 0.9897 | ns | 0.654 | * | 0.0155 | *** | 0.0003 |
| high THCA vs. hybrid | ns | 0.9245 | * | 0.0143 | ns | 0.0567 | * | 0.0294 | **** | <0.0001 | ns | 0.6726 | ns | 0.9806 | ns | 0.7309 | ns | 0.4753 | ns | 0.2062 | * | 0.0119 | ns | 0.5532 |
| significant tests | 0 |  | 2 |  | 1 |  | 2 |  | 3 |  | 2 |  | 0 |  | 0 |  | 0 |  | 0 |  | 2 |  | 2 |  |

**Table S4.** Two-way ANOVA results between chemovar aquagram values at a significance value of p < 0.05. P value < 0.0332 (*), p < 0.0021 (**), p < 0.0002 (***), p < 0.0001 (****).

|  | 1342 nm C1 | | 1364 nm C2 | | 1374 nm C3 | | 1384 nm C4 | | 1412 nm C5 | | 1426 nm C6 | | 1440 nm C7 | | 1452 nm C8 | | 1462 nm C9 | | 1476 nm C10 | | 1488 nm C11 | | 1512 nm C12 | |
| --- | --- | --- | --- | --- | --- | --- | --- | --- | --- | --- | --- | --- | --- | --- | --- | --- | --- | --- | --- | --- | --- | --- | --- | --- |
| Tukey's multiple comparisons test | Summary | P Value | Summary | P Value | Summary | P Value | Summary | P Value | Summary | P Value | Summary | P Value | Summary | P Value | Summary | P Value | Summary | P Value | Summary | P Value | Summary | P Value | Summary | P Value |
| 156 vs. 240 | ns | 0.1626 | ns | 0.1846 | * | 0.0187 | * | 0.0152 | **** | <0.0001 | ns | 0.0984 | ns | 0.9121 | ns | 0.9995 | ns | >0.9999 | ns | 0.9896 | ns | 0.3322 | ** | 0.0051 |
| 156 vs. 45-3 | ns | 0.9533 | **** | <0.0001 | *** | 0.0001 | *** | 0.0005 | ns | 0.7747 | ns | 0.9998 | ns | 0.9939 | ns | 0.7814 | ns | 0.3993 | ns | 0.0623 | *** | 0.0002 | ns | 0.6525 |
| 156 vs. 505 | ns | 0.1773 | ** | 0.0022 | *** | 0.0005 | ** | 0.0011 | **** | <0.0001 | ns | 0.145 | ns | 0.9624 | ns | >0.9999 | ns | >0.9999 | ns | 0.9468 | ns | 0.1277 | ** | 0.0024 |
| 156 vs. 621-17 | * | 0.0139 | ns | 0.9991 | ns | 0.8867 | ns | 0.1515 | ns | 0.4334 | *** | 0.0001 | ** | 0.003 | * | 0.0136 | * | 0.0205 | ns | 0.211 | ns | >0.9999 | * | 0.0158 |
| 156 vs. erez | ns | 0.2851 | **** | <0.0001 | **** | <0.0001 | **** | <0.0001 | **** | <0.0001 | ns | 0.2137 | ns | 0.9976 | ns | 0.9996 | ns | 0.9074 | ns | 0.2371 | **** | <0.0001 | *** | 0.0002 |
| 156 vs. gen12 | ns | 0.4993 | **** | <0.0001 | **** | <0.0001 | **** | <0.0001 | **** | <0.0001 | ns | 0.2133 | ns | >0.9999 | ns | 0.9895 | ns | 0.7482 | ns | 0.14 | **** | <0.0001 | ** | 0.0054 |
| 240 vs. 45-3 | ns | 0.7178 | ns | 0.1675 | ns | 0.7458 | ns | 0.9185 | **** | <0.0001 | ns | 0.1636 | ns | 0.4314 | ns | 0.3801 | ns | 0.276 | ns | 0.2045 | ns | 0.107 | ns | 0.3761 |
| 240 vs. 505 | ns | >0.9999 | ns | 0.6214 | ns | 0.8918 | ns | 0.971 | ns | 0.5672 | ns | >0.9999 | ns | >0.9999 | ns | >0.9999 | ns | >0.9999 | ns | 0.9999 | ns | 0.9968 | ns | 0.9998 |
| 240 vs. 621-17 | **** | <0.0001 | * | 0.0197 | ns | 0.247 | ns | 0.9717 | **** | <0.0001 | **** | <0.0001 | **** | <0.0001 | *** | 0.0004 | ** | 0.0056 | ns | 0.5364 | ns | 0.2673 | **** | <0.0001 |
| 240 vs. erez | ns | >0.9999 | ** | 0.0026 | ns | 0.1811 | ns | 0.5894 | ns | 0.9586 | ns | >0.9999 | ns | 0.9966 | ns | 0.9679 | ns | 0.8583 | ns | 0.5718 | * | 0.0273 | ns | 0.9634 |
| 240 vs. gen12 | ns | 0.993 | * | 0.0184 | ns | 0.149 | ns | 0.1835 | * | 0.0103 | ns | 0.9998 | ns | 0.9594 | ns | 0.8422 | ns | 0.6395 | ns | 0.3997 | ns | 0.0571 | ns | >0.9999 |
| 45-3 vs. 505 | ns | 0.7222 | ns | 0.9891 | ns | >0.9999 | ns | >0.9999 | **** | <0.0001 | ns | 0.2336 | ns | 0.5933 | ns | 0.5429 | ns | 0.4759 | ns | 0.4451 | ns | 0.4275 | ns | 0.2311 |
| 45-3 vs. 621-17 | **** | <0.0001 | **** | <0.0001 | ** | 0.0044 | ns | 0.4178 | ** | 0.004 | **** | <0.0001 | * | 0.0178 | ns | 0.4166 | ns | 0.8886 | ns | 0.9949 | **** | <0.0001 | **** | <0.0001 |
| 45-3 vs. erez | ns | 0.8603 | ns | 0.8686 | ns | 0.9744 | ns | 0.9976 | **** | <0.0001 | ns | 0.3323 | ns | 0.8386 | ns | 0.9289 | ns | 0.9674 | ns | 0.9962 | ns | 0.9995 | ns | 0.0568 |
| 45-3 vs. gen12 | ns | 0.9751 | ns | 0.9953 | ns | 0.9704 | ns | 0.8971 | **** | <0.0001 | ns | 0.3336 | ns | 0.9435 | ns | 0.9855 | ns | 0.9951 | ns | 0.9993 | ns | >0.9999 | ns | 0.3863 |
| 505 vs. 621-17 | **** | <0.0001 | **** | <0.0001 | * | 0.0124 | ns | 0.5657 | **** | <0.0001 | **** | <0.0001 | **** | <0.0001 | ** | 0.002 | * | 0.0231 | ns | 0.8095 | ns | 0.0871 | **** | <0.0001 |
| 505 vs. erez | ns | >0.9999 | ns | 0.3998 | ns | 0.9051 | ns | 0.9865 | ns | 0.9871 | ns | >0.9999 | ns | 0.9996 | ns | 0.9909 | ns | 0.9555 | ns | 0.8263 | ns | 0.1836 | ns | 0.9979 |
| 505 vs. gen12 | ns | 0.9913 | ns | 0.7848 | ns | 0.8921 | ns | 0.7946 | ns | 0.7346 | ns | >0.9999 | ns | 0.9881 | ns | 0.9304 | ns | 0.8319 | ns | 0.6934 | ns | 0.3123 | ns | 0.9997 |
| 621-17 vs. erez | **** | <0.0001 | **** | <0.0001 | **** | <0.0001 | ns | 0.1226 | **** | <0.0001 | **** | <0.0001 | **** | <0.0001 | * | 0.0262 | ns | 0.2946 | ns | >0.9999 | **** | <0.0001 | **** | <0.0001 |
| 621-17 vs. gen12 | **** | <0.0001 | **** | <0.0001 | **** | <0.0001 | * | 0.0147 | **** | <0.0001 | **** | <0.0001 | *** | 0.0001 | ns | 0.0532 | ns | 0.4439 | ns | >0.9999 | **** | <0.0001 | **** | <0.0001 |
| erez vs. gen12 | ns | 0.9994 | ns | 0.9942 | ns | >0.9999 | ns | 0.9965 | ns | 0.2229 | ns | >0.9999 | ns | 0.9999 | ns | 0.9999 | ns | >0.9999 | ns | >0.9999 | ns | 0.9998 | ns | 0.9601 |
| significant tests | 6 |  | 11 |  | 9 |  | 6 |  | 14 |  | 6 |  | 6 |  | 4 |  | 3 |  | 0 |  | 7 |  | 10 |  |

**
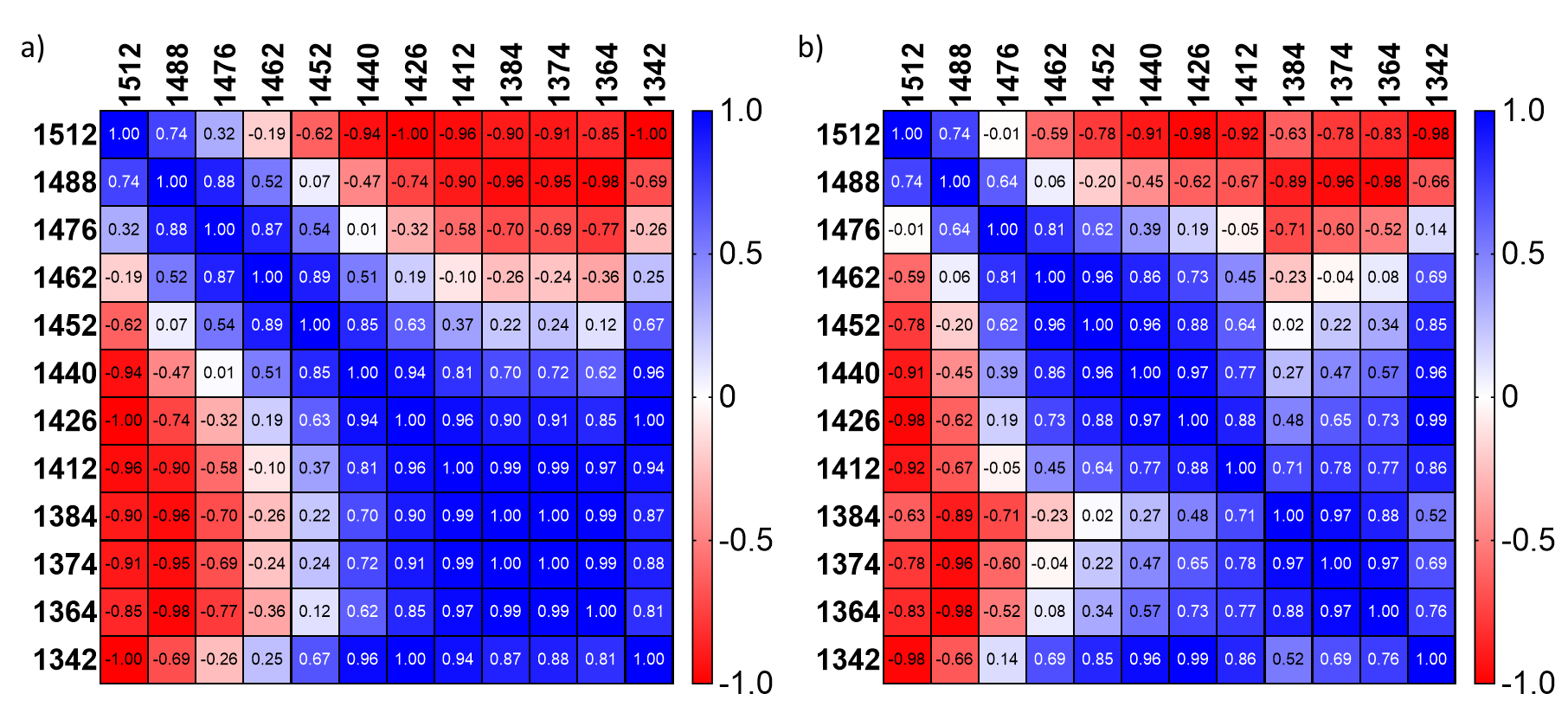
**

**Figure S1.** Pearson r correlation coefficients matrix values of (a) major-class and (b) chemovar aquagram WAMACS values.

**Table S5.** Average concentrations of major cannabinoids and terpenes (± standard deviation, normalized to DW% using the water content of each chemovar as detailed in Table S1) and wet water content percentage for each chemovar.

| **Chemovar** | **621-17** | **Erez** | **505** | **240** | **Gen12** | **156** | **45-3** |
| --- | --- | --- | --- | --- | --- | --- | --- |
| **Wet water content** | 79.7 ± 0.4 | 75 ± 3 | 78.5 ± 2.0 | 78.5 ± 1.6 | 77.4 ± 1.6 | 76.7 ± 1.0 | 73.4 ± 2.1 |
| **THCA** | 17.0 ± 1.3 | 14.7 ± 3.0 | 19.5 ± 3.8 | 9.2 ± 1.5 | 4.5 ± 0.7 | 0.28 ± 0.09 | 0.29 ± 0.07 |
| **CBDA** | 0.04 ± 0.01 | 0.09 ± 0.02 | 0.06 ± 0.01 | 0.07 ± 0.01 | 10.0 ± 1.5 | 11.5 ± 0.9 | 6.3 ± 1.6 |
| **CBGA** | 0.30 ± 0.02 | 1.1 ± 0.3 | 1.0 ± 0.2 | 0.4 ± 0.2 | 0.8 ± 0.2 | 0.26 ± 0.06 | 0.14 ± 0.04 |
| **CBCA** | 0.27 ± 0.05 | 0.26 ± 0.07 | 0.21 ± 0.04 | 0.15 ± 0.03 | 0.58 ± 0.14 | 0.46 ± 0.10 | 0.34 ± 0.09 |
| **α-pinene** | 0.10 ± 0.02 | 0.14 ± 0.03 | 0.13 ± 0.02 | 0 | 0.32 ± 0.06 | 0.11 ± 0.02 | 0.16 ± 0.06 |
| **(-)-β-pinene** | 0.13 ± 0.03 | 0.06 ± 0.01 | 0.10 ± 0.02 | 0.011 ± 0.003 | 0.15 ± 0.03 | 0.055 ± 0.008 | 0.05 ± 0.02 |
| **β-myrcene** | 0.09 ± 0.03 | 0.9 ± 0.2 | 0.026 ± 0.003 | 0.06 ± 0.01 | 0.43 ± 0.08 | 0.74 ± 0.14 | 0.06 ± 0.02 |
| **d-limonene** | 0.48 ± 0.13 | 0.08 ± 0.02 | 0.38 ± 0.06 | 0.09 ± 0.02 | 0.08 ± 0.02 | 0.057 ± 0.009 | 0.006 ± 0.006 |
| **β-caryophyllene** | 0.38 ± 0.14 | 0.13 ± 0.03 | 0.25 ± 0.04 | 0.46 ± 0.07 | 0.59 ± 0.08 | 0.19 ± 0.04 | 0.06 ± 0.02 |
| **α-humulene** | 0.15 ± 0.05 | 0.06 ± 0.02 | 0.07 ± 0.01 | 0.19 ± 0.03 | 0.19 ± 0.03 | 0.04 ± 0.01 | 0.01 ± 0.01 |
| **(-)-guaiol** | 0.07 ± 0.02 | 0.04 ± 0.01 | 0.051 ± 0.008 | 0.08 ± 0.01 | 0.07 ± 0.01 | 0.10 ± 0.02 | 0.03 ± 0.01 |
